# Childhood-onset primary Sjögren’s syndrome in a tertiary center in China: clinical features and outcome

**DOI:** 10.1186/s12969-022-00779-3

**Published:** 2023-01-27

**Authors:** Yinv Gong, Haimei Liu, Guomin Li, Tao Zhang, Yifan Li, Wanzhen Guan, Qiaoqian Zeng, Qianying Lv, Xiaomei Zhang, Wen Yao, Yu Shi, Hong Xu, Li Sun

**Affiliations:** Department of Rheumatology, Children’ Hospital of Fudan University, National Children’s Medical Center, No. 399 Wanyuan Road, Shanghai, 201102 China

**Keywords:** Primary Sjögren's syndrome, Childhood, Diagnosis, Clinical features, Outcome

## Abstract

**Objectives:**

To characterize the clinical features and outcomes of childhood-onset primary Sjögren’s syndrome (pSS).

**Methods:**

Patients less than 18 years old who were diagnosed with pSS by paediatric rheumatologists were included, and all patients were applied the 2002 American-European Consensus Group (ACEG) criteria, the 2016 American College of Rheumatology/European League Against Rheumatism (ACR/EULAR) criteria for pSS, or the 1999 proposed juvenile pSS criteria. The electronic medical records of patients with pSS from 2013 to 2020 were collected and analysed.

**Results:**

Thirty-nine patients were included. Of them, 27 (69.2%), 38 (97.4%) and 35 (89.7%) patients fulfilled the AECG criteria, ACR/EULAR criteria and proposed juvenile pSS criteria, respectively. The female:male ratio was 3.9:1. The median ages at first signs or symptoms and at diagnosis were 9.2 (4.7, 14.5) years and 10.9 (6.3, 15.0) years, respectively. The main clinical manifestations were rash or purpura (20, 51.3%), followed by fever (12, 30.8%), glandular enlargement/recurrent parotitis (10, 25.6%), and dry mouth and/or dry eyes (9, 23.1%). Twenty-eight (56.4%) patients had systemic damage, the most common of which was haematological involvement (14, 35.9%), followed by hepatic (13, 33.3%) and renal involvement (8, 20.5%). Thirty-eight (97.4%) patients underwent labial minor salivary gland biopsy, and all exhibited focal lymphocytic sialadenitis. All patients had a global ESSDAI score ≥ 1 at diagnosis, and the median total score at diagnosis was 8 (2, 31). Thirty-six (92.3%) patients were followed up for a median time of 23.6 (7.9, 79.5) months, and three patients developed systemic lupus erythematosus (SLE) at follow-up times of 13.3, 38.8 and 63.8 months.

**Conclusions:**

The presentation of childhood-onset pSS is atypical, and extraglandular manifestations and systemic involvement are more common than in adult-onset pSS. Labial salivary gland biopsy is vital for patients with probable pSS. Some patients may develop SLE over time.

**Supplementary Information:**

The online version contains supplementary material available at 10.1186/s12969-022-00779-3.

## Introduction

Primary Sjögren’s syndrome (pSS) is a systemic autoimmune disease, predominantly affects middle-aged women, with a frequency ranging between 0.01 and 0.72% [1]. Patients with pSS mainly experience oral and ocular dryness and extraglandular manifestations (EGM), including interstitial pneumonitis, interstitial nephritis, isosthenuria or renal tubular acidosis, thyroiditis, central nervous system involvement, vasculitis, and an increased incidence of lymphoma [2].

In children, pSS is less well characterized in terms of clinical presentation and long-term outcomes [3]. When the disease starts before the age 18 of years, it is called SS with childhood onset or juvenile SS. Childhood-onset pSS is rarely reported, poorly defined and possibly underdiagnosed due to the different criteria used for diagnosis and the scarcity of available data [4]. A recent report of the Sjogren Big Data Consortium revealed that childhood-onset pSS involved approximately 1% of patients with pSS, with a clinical phenotype dominated by sicca features, parotid enlargement, and the highest frequencies of systemic disease in 5 (constitutional, lymphadenopathy, glandular, cutaneous and haematological) of the 12 European League Against Rheumatism (EULAR) Sjögren's syndrome disease activity index (ESSDAI) domains [5].

Neither of the currently most widely used classification criteria of pSS, the American-European Consensus Group (AECG) criteria [6] and the American College of Rheumatology/EULAR criteria [7], have been validated in a juvenile population. Child-specific criteria have been proposed [8]; however, even these criteria have a sensitivity of only 76% for diagnosing childhood SS [9, 10].

In the absence of established classification criteria specific for pSS in children, we chose to focus our study on children who met any of the current classification criteria [6, 7, 8]. Therefore, the aim of this study was to characterize the clinical features, immunological profiles and long-term outcomes of childhood-onset pSS in a cohort of Chinese patients from a tertiary paediatric medical centre.

## Methods

### Patients

In this retrospective longitudinal cohort study, patients who were diagnosed with pSS by paediatric rheumatologists with an onset age of 18 years or younger were included. Patients who were suspected of having secondary SS were excluded. At the time of inclusion, all patients were applied the AECG criteria [6], the ACR/EULAR criteria [7], or the 1999 proposed diagnostic criteria for juvenile pSS [8]. The electronic medical records of patients with new-onset pSS from the Children’s Hospital of Fudan University, Shanghai, China, between January 2013 and December 2020 were screened. Children’s Hospital of Fudan University is one of the three National Children's Hospital centres, and all data collected were reliable and complete. The following clinical variables were selected for analysis:

- Epidemiological features: age (age at onset and at diagnosis) and gender.
- Clinical features: disease course and initial and main symptoms and/or signs suggesting an autoimmune disease.
- Systemic activity: systemic involvement at diagnosis was classified and scored according to the EULAR ESSDAI [11]. The haematological and biological domains were determined from the most recent blood samples available.
- Immunological profile: antinuclear antibodies (ANA), anti-Ro(SSA)/La(SSB) antibodies, complement C3 and C4 levels, anti-double strand DNA antibody (anti-dsDNA antibody), serum immunoglobulin G (IgG), rheumatoid factor (RF) and erythrocyte sedimentation rate (ESR).
- Histological data: labial minor salivary gland (LSG) biopsy.
- Outcome (for patients followed for more than 1 year): developed to SLE or progressed to Lymphoma.

The observation end point was the last follow-up record in our centre.

### Ethical considerations

The study was approved by the Research Ethics Board of Children’s Hospital of Fudan University and carried out in accordance with the guidelines of the Declaration of Helsinki. Patients who agreed to participate gave their written informed consent.

### Statistical analysis

Descriptive data are presented as medians (ranges) for enumerated data and numbers and percentages (%) for categorical variables. The chi-square test was used to compare categorical variables. All analyses were performed using IBM SPSS Statistics version 26.0 (Armonk, NY, IBM Corp).

## Results

### Patient characteristics

Our cohort consisted of 39 patients. The female:male ratio was 3.9:1 (31:8). The median age at the first sign or symptom suggestive of the disease and at the time of diagnosis of pSS were 9.2 (4.7, 14.5) years and 10.9 (6.3,15.0) years, respectively. The time interval from onset to diagnosis was 6.5 (0.4, 72.9) months (Table 1).

**Table 1:** Epidemiological features and criteria fulfillment of 39 patients with primary Sjögren’s syndrome diagnosed in childhood

Variable	Patients diagnosed with childhood-onset pSS (*n* = 39)
Gender and age	
Female(%)	31(79.5)
Age at diagnosis (yrs)/Median(range)	10.9 (6.3, 15.0)
Age at initial sign/symptom (yrs)/Median(range)	9.2 (4.7, 14.5)
Diagnosis delayed time (mths) /Median(range)	6.5 (0.4, 72.9)
Classification criteria	
AECG criteria	27 (69.2)
ACR/EULAR criteria	38 (97.4)
Proposal juvenile pSS criteria	35 (89.7)
AECG or ACR/EULAR	38 (97.4)
AECG or Proposal juvenile pSS criteria	37 (94.9)
ACR/EULAR or Proposal juvenile pSS criteria	39 (100)

Out of the 39 patients, 27 (69.2%), 38 (97.4%) and 35 (89.7%) fulfilled the AECG criteria, ACR/EULAR criteria and proposed juvenile pSS criteria, respectively. Thirty-eight (97.4%) patients fulfilled either the AECG or ACR/EULAR criteria, 37 (94.8%) patients fulfilled either the AECG criteria or the proposed juvenile pSS criteria, and all patients fulfilled either the ACR/EULAR or the proposed juvenile pSS criteria (Table 1).

### Clinical features

In our cohort, the main clinical manifestations were skin involvement [20 (51.3%), including rash (erythema, urticaria, etc.) (12, 30.8%), and purpura (8, 20.5%)], followed by fever (12, 30.8%), glandular enlargement/recurrent parotitis (10, 25.6%), and dry mouth and/or dry eyes (9, 23.1%). The main manifestations of pSS diagnosed in childhood are summarized in Table 2.

**Table 2:** Clinical features of 39 patients with primary Sjögren’s syndrome diagnosed in childhood

Symptoms & signs	**N(%)**
Glandular enlargement/recurrent parotitis	10 (25.6)
Dry mouth + dry eyes	2 (5.1)
Isolated dry mouth or dry eyes	7 (17.9)
Fever	12 (30.8)
Arthralgia	7 (17.9)
Arthritis	1 (2.6)
Skin involvement	20 (51.3)
Rash	12 (30.8)
Purpura	8 (20.5)
Hematological involvement	14 (35.9)
Leukopenia	7 (17.9)
Thrombocytopenia	6 (15.4)
Anemia	4 (10.3)
Hepatic involvement	13 (33.3)
Lymphadenopathy	9(23.1)
Renal involvement	8 (20.5)
RTA	4 (10.3)
Proteinuria	4 (10.3)
Renal impairment	3 (7.7)
Interstitial pneumonia	3 (7.7)
Neurological involvement	1 (2.6)
Hypokalemia	4 (10.3)
Fatigue	1 (2.6)
Raynaud phenomenon	1 (2.6)

**Fig. 1:**
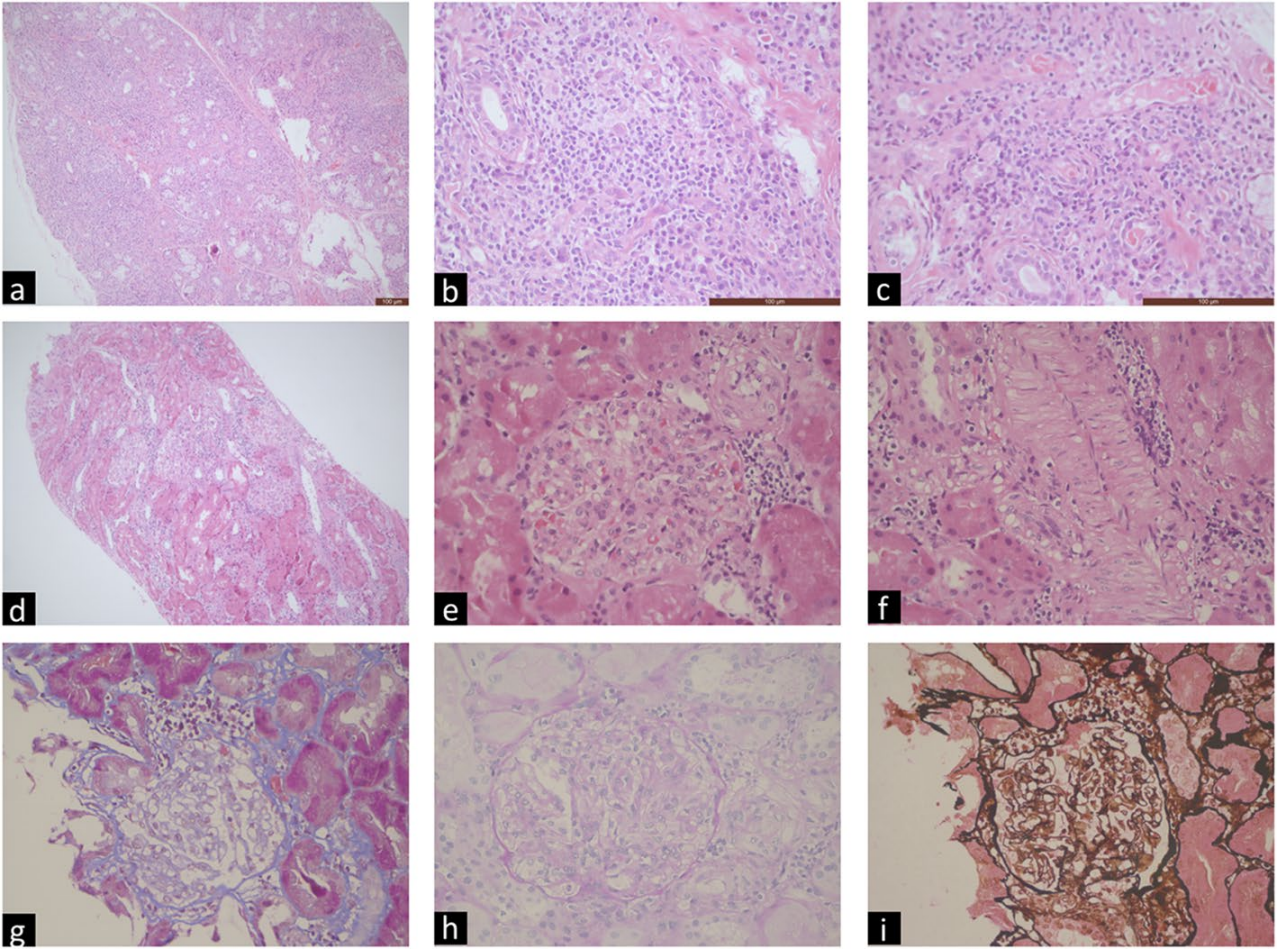
Histological findings in the patient with primary Sjögren ‘s syndrome with cholestasis, tubulointerstitial nephritis and 47,XXX. **a**-**b**: multifocal lymphocytic infiltrates with periductal distribution in lacrimal salivary gland biopsy section. Magnifications: × 100 in a and × 400 in b, hematoxylin and eosin, bar size: 100 µm; **d**-**i**: focal proliferative glomerulonephritis and mild tubulointerstitialitis in renal biopsy section. Magnifications: × 100 in d (hematoxylin and eosin), × 400 in e and f (hematoxylin and eosin), × 400 in g (masson), × 400 in h (periodic acid schiff) and × 400 in h (periodic acid-silver methenamine)

Twenty-eight (56.4%) patients presented with essential systemic damage, the most common of which was haematological involvement [14 (35.9%), including leukopenia (7,17.9%), thrombocytopenia (6, 15.4%), and anaemia (4,10.3%)], followed by hepatic involvement [13 (33.3%), mainly manifested as elevated liver enzymes, especially one patient with cirrhosis and cholestasis] and renal involvement [8 (20.5%), including renal tubular acidosis (RTA) (4, 10.3%), proteinuria (4, 10.3%) and renal impairment (3, 7.7%)]. Of them, one patient initially manifested with elevated leucocytes and jaundice and was diagnosed with pSS combined with cholestasis, RTA, proteinuria [renal biopsy confirmed focal proliferative glomerulonephritis with mild tubulointerstitial nephritis (TIN)], lower limb peripheral neuropathy and 47,XXX (Fig. 1). In addition, the initial symptom/sign at onset in one patient was uveitis, not dry eye. Three patients had interstitial pneumonia (Table 2).

In total, four patients underwent renal biopsy, one of whom was described above. In the second patient, the initial symptom/sign at onset was Raynaud phenomenon and arthralgia combined with ANA and SSA positivity and low C3 levels, and the renal pathological result was focal proliferative glomerulonephritis, differentiating it from SLE. The last two patients were confirmed to have lupus nephritis by biopsy when suspected of having SLE during the follow-up period.

### Diagnostic tests, immunological markers and systemic activity

With respect to the diagnostic approach, abnormal ocular tests were reported in 24.3% (9/37) of those studied using corneal fluorescein staining, and abnormal oral tests were reported in 81.5% (22/27) of those studied by parotid scintigraphy. Unfortunately, we could not evaluate Schirmer’s test, conjunctival lissamine green staining, or unstimulated whole salivary flows. Thirty-eight (97.4%) patients underwent LSG biopsy, and all exhibited focal lymphocytic sialadenitis with a focus score of ≥ 1 focus/4 mm^2^ (Table 3).

**Table 3:** Diagnostic tests, immunological markers and systemic activity at the time of diagnosis

Variable	Patients diagnosed with childhood-onset pSS (*n* = 39)
DIAGNOSTIC TESTS	
Abnormal ocular tests (any)	
Schirmer's test	NE
Rose bengal score/other ocular dye score	9 (23.1)
Abnormal oral diagnostic tests (any)	
Unstimulated whole salivary flow	NE
Parotid sialography	NE
Salivary scintigraphy	22 (81.5)^a^
Positive minor salivary gland biopsy	38 (100)^b^
IMMUNOLOGICAL PROFILE	
Positive anti-SSA/SSB antibodies	39 (100)
Anti-SSA antibodies	39 (100)
Anti-SSB antibodies	18 (46.1)
ANA	37 (94.9)
Anti-dsDNA antibody	0
Low C3 levels	4 (10.3)
Low C4 levels	2 (5.1)
RF-positive	17 (43.6)
Elevated ESR	27 (69.2)
Elevated IgG	35 (89.7)
SYSTEMIC ACTIVITY	
ESSDAI score/ Median(range)	8 (2, 31)
ESSDAI domains (score > = 1)	
Constitutional	11 (28.2)
Lymphadenopathy	9 (23.1)
Glandular	8 (20.5)
Articular	4 (10.3)
Cutaneous	14 (35.9)
Pulmonary	3 (7.7)
Renal	5 (12.8)
Muscular	0
PNS	1 (2.6)
CNS	0
Hematological	11 (28.2)
Biological	31 (79.5)

^a^ Twelve patients didn’t complete Salivary scintigraphy

^b^ One patient didn’t complete minor salivary gland biopsy

*SS* Sjögren syndrome, *ESSDAI* EULAR Sjögren's syndrome disease activity index, *ESR* Erythrocyte sedimentation rate, *IgG* Immunoglobulin G, *PNS* Peripheral nervous system, *CNS* Central nervous system, *NE* Not evaluated

For the immunological profile at the time of diagnosis, the most frequent abnormalities consisted of anti-Ro/SSA antibody positivity (39, 100%), ANA positivity (37, 94.9%), polyclonal hyperimmunoglobulinaemia (35, 89.7%), and a high ESR (27, 69.2%). None of the patients were positive for anti-dsDNA antibodies (Table 3).

Regarding systemic phenotype, the median total ESSDAI score at diagnosis of our cohort was 8 (2, 31), and all patients had systemic activity at diagnosis (global ESSDAI score ≥ 1). According to the EULAR definition, 8 patients had inactive disease (ESSDAI score < 5) at diagnosis. The frequently involved ESSDAI domains at diagnosis were biological (79.5%), followed by the cutaneous (35.9%), constitutional (28.2%), haematological (28.2%) and glandular (20.5%) domains. The distribution of the ESSDAI scores for the entire cohort for each domain is summarized in Supplementary Table S1.

### Therapeutic regime

Hydroxychloroquine (HCQ) was prescribed to all patients in our cohort, and three of them were treated with HCQ alone. Regarding the treatment regimen given at the time of diagnosis, 36 patients were prescribed oral glucocorticoids (GCs), and 12 (30.7%) of them were prescribed mycophenolate mofetil (MMF) initially. Six patients on GCs and HCQ were prescribed MMF due to disease flares or relapse during GC tapering or spontaneous withdrawal of GCs. MMF was given as maintenance therapy to four patients using CYC, and a substitute of CsA was given to one patient with immune haemolytic anaemia and thrombocytopenia when the disease was controlled. A total of 23 (58.9%) patients were prescribed MMF (Supplement Table S1).

### Outcome

Thirty-six (92.3%) patients were followed up for a median time of 23.6 (7.9, 79.5) months, and 3 patients were lost to follow-up. Thirty-one (79.4%) patients were followed up for more than 1 year. Three patients developed SLE at a median follow-up time of 38.8 months. One patient was a 13.3-year-old boy with persistent ANA positivity, emerging proteinuria, low complement and anti-Sm antibody positivity who was diagnosed by renal biopsy with lupus nephritis (LN) II at 13.3 months. The second patient was an 11.6-year-old boy who was diagnosed with SLE based on high titres of ANAs and anti-dsDNA antibody, myocarditis and pericardial effusion, and acute cutaneous lupus presented as a photosensitive lupus rash at 38.8 months. The third patient was an 11.7-year-old girl with persistent high titres of ANAs, emerging low complement, anti-Sm antibody and anti-dsDNA antibody positivity, and mild proteinuria and was then diagnosed by renal biopsy with LN III at 63.8 months. No patient had progressed to lymphoma at the last follow-up.

With respect to the other 28 patients, 26 of them achieved a good response, only two patients still experienced mild rash relapse, and no patients exhibited moderate or severe disease activity. Regarding GC tapering, 11 patients withdrew GCs at a median time of 18 (15, 25) months, 13 patients maintained a small dose of GCs (< 5 mg/d) and tapered to 5 mg/d at a median time of 14 (8, 25) months, two patients persistently did not use GCs, and one patient stopped GCs by herself. One patient tapered GCs to a dose of 7.5 mg/d.

## Discussion

The number of studies on and the number of reported cases of childhood-onset pSS are very limited. The data available to date suggest that pSS is a very rare disease in children. Manuel Ramos-Casals et al. [5] estimated that the frequency of childhood-onset pSS was approximately 1% of patients with primary SS based on the worldwide data-sharing cooperative merging of preexisting clinical SS databases from the five continents, which was to date the largest multicentre study investigating the clinical characteristics of childhood-onset pSS, confirming that pSS is a disease that is rarely diagnosed in children.

Currently available classification criteria [6, 7] have been developed for adult patients, but they are not well validated in childhood-onset SS. Houghton K et al. [9] reported that 39% of patients met the AECG criteria and 76% met the proposed paediatric criteria; the authors proposed that the AECG adult classification criteria for pSS should not be applied to children as the sensitivity is unacceptably low. In addition, a recent study by Basiaga ML et al. [12] showed that the majority of children diagnosed with SS in an international childhood SS cohort did not meet the 2016 ACR/EULAR classification criteria, and the authors emphasized the need for further research, including the creation of paediatric-specific classification criteria. In our cohort, we comprehensively evaluated the existing adult criteria as well as the proposed juvenile pSS criteria [8], 97.4% of patients fulfilled the ACR/EULAR criteria, 89.7% fulfilled the proposed juvenile pSS criteria and 69.2% fulfilled the AECG criteria. The ACR/EULAR criteria are applicable to any patient with at least one symptom of ocular or oral dryness and consider systemic signs and B-cell activation biomarkers (using the ESSDAI) as inclusion criteria [7], which allows the diagnosis of systemic and earlier forms of the disease when sicca features are not already present. All but one patient (97.4%) in our series had systemic activity at diagnosis (global ESSDAI score ≥ 1) and was diagnosed with SS according to the presence of SSA autoantibodies and LSG biopsy findings, and the last patient’s diagnosis of SS was made according to the proposed juvenile pSS criteria. The ACR/EULAR criteria performed best in our study and might be optimal for the early diagnosis of and clinical research on childhood-onset pSS when combined with the proposed juvenile pSS criteria.

Based on the findings of previous studies [5, 13, 14, 15, 16, 17], the clinical glandular phenotype of childhood-onset SS is mainly dominated by parotid involvement rather than by sicca features, and the symptoms of dry mouth and eyes mostly appear during the course of the disease and rarely as the first complaint. Data from the Sjögren Big Data Consortium [5, 18] confirm that the systemic phenotype of pSS at diagnosis is strongly influenced by personal determinants such as age, gender, and ethnicity. They revealed that patients with childhood-onset pSS showed the highest mean ESSDAI score and the highest frequencies of systemic disease in 5 (constitutional, lymphadenopathy, glandular, cutaneous and haematological) of the 12 ESSDAI domains in comparison with patients with adult-onset disease. In adults, Black/African American patients had the highest frequencies of activity in the lymphadenopathy, articular, neurological and biological domains; White patients in the glandular, cutaneous and muscular domains; Asian patients in the pulmonary, renal and haematological domains; and Hispanic patients in the constitutional domains. More than half of the patients in our study presented with EGMs, and skin, haematological, hepatic and renal involvement were more predominant than in other studies. As summarized in Table 4, ethnic differences might also play a role.

**Table 4:** Summary of characteristics of childhood onset SS

Authors	Tomiita, M et al. [17]	Cimaz, R et al. [16]	Yokogowa, N et al. [14]	Hammenfors, D.S et al. [13]	Ramos-Casals, M et al. [5]	Basiaga, M. L et al. [12]	Our data
Data of country	Japan nationwide	Multi-center	USA	Multi-center	Multi-center	International	China
Published year (Study year)	1997 (1994.7–1995.4)	2003 (1998–2000)	2016	2020	2021	2021	Present study
No. cases	42	40	26	67	158	300	39
Female: male ratio	/	7:1	12:1	6.4:1	6.2:1	5:1	3.9:1
Age of onset (yrs)^a^	10.7 (3, 15)	10.7	/	10.2 (1, 17)	13.2	/	9.5 (4.7, 14.5)
Age of diagnosis (yrs) ^a^	/	12.4	12.3 (4,17.8)	12.1 (4, 18)	14.2	12(1, 17.8)	10.8 (6.3, 15.0)
Time-interval from onset to diagnosis (mths)^a^	/	/	/	/	/		4.4 (0.4, 72.9)
Recurrent parotitis	35.7	72.5	65.3	63.8	35.8	46.7	25.6
Dry eyes	6^b^	12.5	65.3	62.6	79.7	48.0	10.4
Dry mouth	/	12.5	65.4	80.3	70.2	52.0	17.9
Extra-glandular manifestation							
Fever	52.3	10.0	23.0	/	11.9	11.3	**30.8**
Skin	26.1	/	15.4	28.8	9.3	9.0	**51.3**
Arthralgia	28.6	10.0	57.7	57.6	9.9	53.6	**17.9**
Hematological	/	/	15.4	27.6	2.6	17.3	**35.9**
Hepatic	2.4	10.0	/	/	/	/	**33.3**
Renal	7.1	7.5	19.2	5.1	4.5	9.0	**20.5**
Pulmonary	2.4	/	/	1.7	5.2	8.3	**7.7**
Neurological	7.1	5.0	23.1	5.1	0.6	11.3	**2.6**
Lymphadenopathy	26.2	7.5	42.3	59.3	25.2	18.0	**23.1**
Immunological profile							
anti-SSA or SSB	/	73.6	84.6	74.6	88.5	97.3	**100**
anti-SSA	61.9	/	84.6	74.6	82.7	97.3	**100**
anti-SSB	31.0	/	65.4	40.3	61.9	97.0	**46.1**
Positive ANA	81.0	85	96.2	92.5	90.3	99.7	**94.9**
Elevated IgG	76.2	53.1	/	/	/	76.7	**89.7**
Positive RF	57.1	75.0	72.7	45.3	67.6	85.7	**43.6**
Elevated ESR	/	68.4		/	/	/	69.2
Labial salivary gland biopsy (No. positive/No.performed)	69.0%	100%	100% (15/15)	79.3% (23/29)	96.7% (118/122)	53.4% (70/131)	**100%** **(38/38)**

^a^ median (range)

^b^ including dry eye and/or dry mouth; yrs: years old; mths: months; /: not available

It is remarkable that one-third of patients in our cohort had abnormal hepatic biochemical indices, while only a few studies have reported the frequency (2.4–10.0%) of abnormal hepatic biochemical indices in children [16, 17]. Hepatic involvement is observed in 27 to 49% of cases of pSS in adults, and half of these patients have overt clinical liver disease. The most common liver diseases are primary biliary cholangitis (PBC) and autoimmune hepatitis (AIH) [19, 20]. Hepatic involvement is also frequently found in patients with childhood-onset pSS; therefore, children with pSS should be monitored to identify clinical liver disease early. Interestingly, one patient in our series was diagnosed with pSS combined with cholestasis, RTA, proteinuria (focal proliferative glomerulonephritis and mild TIN), lower limb peripheral neuropathy and 47,XXX. 47,XXX is a rare event and is significantly increased among patients with SLE and SS. The estimated prevalence of SS with 47,XXX in women was 0.29%, which was 2.9 times and 41 times higher than that in women with 46,XX and that in men with 46,XY [21]. This case supported the link between the presence of extra X chromosome(s) and the development of autoimmune diseases and enriched the phenotype of 47,XXX with autoimmune diseases.

Renal involvement in SS is rare, affecting < 10% of patients (range from ~ 1 to > 30% in different studies and ethnicities of adult patients [22] and 4.5–19.2% in paediatric patients). Renal involvement in pSS is heterogeneous and ranges from isolated electrolyte disturbances and nephrolithiasis to glomerulonephritis and TIN in both acute and chronic forms, and TIN is the most frequent renal complication of pSS. One-fifth of our case series had renal involvement, and four patients underwent renal biopsy (one was to confirm the severity of TIN, the second was to differentiate with SLE, and the last two were confirmed to have lupus nephritis by biopsy when SLE was suspected during the follow-up period). Kidney biopsy can confirm the severity of TIN to determine the treatment regimen and help in the differential diagnosis, especially when glomerular disease is suspected. A kidney biopsy should be performed as soon as possible to rule out other possible causes, such as lupus nephritis.

In addition, in view of ocular involvement, uveitis should be considered as well, similar to earlier reports [17, 23], and deserves comprehensive and continuously monitored systemic evaluation. SS can cause serious, vision-threatening extraglandular ocular manifestations in addition to dry eye.

When childhood pSS is suspected clinically, an appropriate workup should be performed. With respect to the study of exocrine gland dysfunction, some diagnostic tests used in adults are not validated in children [12], such as the Schirmer test, unstimulated whole saliva flows, and conjunctival lissamine green staining, due to the limited cooperation of the children and the unavailability of stains. We performed corneal fluorescein staining to determine the presence of lacrimal gland disease, and positive findings were noted in only nine (24.3%) of the 37 children evaluated, suggesting that positive ocular staining may be a later manifestation, consistent with the clinical features of sicca symptoms. The finding of focal lymphocytic sialadenitis on minor labial salivary gland biopsy is specifically suggestive of childhood pSS [24, 25]. In previous studies [5, 13, 14, 15, 16, 17], salivary gland biopsy was positive in 53–100% of children (Table 4), and the frequency in our study increased to 100% among the 97.4% of patients who underwent biopsy, which could explain the high rate of meeting the EUALR criteria in our study, while only 43.6% of children in the study of Basiaga ML et al. underwent LGS biopsies, and nearly half exhibited positive findings [12]. Histopathological analyses should also include the biopsy of other salivary or lacrimal glands if tissue was procured for other purposes, such as to rule out lymphoma.

Salivary gland ultrasound (SGUS) is a noninvasive test that may be used to evaluate the glandular damage of salivary glands in children with Sjögren’s syndrome [26]. Recently, Hammenfors et al. [13] reported pathological SGUS findings in 41 (61%) of 67 patients with childhood-onset SS and indicated that SGUS findings were associated with hyposalivation and autoantibodies. In the study of Manuel Ramos-Casals [5], the rate was even higher (94%). In our centre, although five of ten patients with parotid involvement underwent SGUS, abnormal findings were revealed in 100%. SGUS might be an interesting diagnostic tool for identifying patients with pSS in childhood, and further clinical application is needed.

The majority of our patients achieved a good response at a median follow-up time of nearly two years, while three patients (nearly 10%) had SLE at 13.3 months, 38.8 months and 63.8 months. Previous studies published that 7.5–10% of patients with long-standing pSS eventually developed a systemic disorder that fulfilled the criteria for SLE in adults [27, 28]. Similar to paediatric patients, Schuetz C et al. [10] reported that one in eight (12.5%) children with pSS developed overlapping lupus nephritis one year after the diagnosis of pSS. The coexistence of SS and SLE was first demonstrated in 1959 [29]. Compared with SLE alone, this combined disease of SS-SLE has distinct features with relatively less major internal organ involvement but a more specific immunological profile and more favourable clinical outcomes [30]. Yujiao Y et al. revealed that patients with Sjögren's syndrome that progresses to systemic lupus erythematosus (SS/SLE) had an earlier age of onset and higher incidences of proteinuria and low complement levels than patients with pSS in a retrospective case‒control study. Two of them experienced hypocomplementemia and lupus nephritis; thus, long-term follow-up and careful evaluation for SLE in patients with childhood-onset pSS are recommended, and we should be vigilant with patients with these mentioned clinical and laboratory characteristics, who have a higher risk of developing SS/SLE. No patients in our cohort progressed to lymphoma in a median follow-up of nearly 2 years. The complication of lymphoma is rare but is reported in some children with pSS [31, 32]. Whether the risk is similar to or greater than that in adults is not yet known, and attention to this complication is needed in children with pSS.

The study has some limitations. As this was a retrospective study, we analysed preexisting data obtained from electronic medical records; therefore, recall bias was inevitable. This was a single-centre study of Chinese children, and a larger cohort size based on a multicentre study or national registry system is necessary to better define and understand the natural history of pSS in children and to determine which features accurately predict a diagnosis and progression.

In conclusion, the main presentation of childhood-onset pSS were atypical, and extraglandular manifestations and systemic involvement were more common. Labial salivary gland biopsy is vital for patients with probable pSS. The 2016 ACR/EULAR criteria might be optimal for early diagnosis based on systemic assessment and labial salivary gland biopsy. Some patients with typical pSS may develop SLE over time, and attention is drawn to close monitoring and long-term follow-up.

## Supplementary Information

**Additional file 1:** **Supplement Table S1.** The distribution of the ESSDAI score for each domain of 39 patients with childhood-onset primary Sjögren’s syndrome.

**Additional file 2.**

## Data Availability

All data generated or analysed during this study are included in this published article and its supplementary information files.

## Abbreviations

pSS: Primary Sjögren’s syndrome
AECG: The American-European Consensus Group
ACR/EULAR: The American College of Rheumatology/European League Against Rheumatism
SLE: Systemic lupus erythematosus
EGM: Extraglandular manifestations
ESSDAI: Sjögren's syndrome disease activity index
ANA: Antinuclear antibodies
anti-dsDNA antibody: Anti-double strand DNA antibody
IgG: Immunoglobulin G
RF: Rheumatoid factor
ESR: Erythrocyte sedimentation rate
LSG: Labial minor salivary gland
RTA: Renal tubular acidosis
TIN: Tubulointerstitial nephritis
HCQ: Hydroxychloroquine
GC: Glucocorticoid
MMF: Mycophenolate mofetil
CYC: Cyclophosephamide
LN: Lupus nephritis
PBC: Primary biliary cholangitis
AIH: Autoimmune hepatitis
SGUS: Salivary gland ultrasound
