# Supplementary material for: Childhood-onset primary Sjögren’s syndrome in a tertiary center in China: clinical features and outcome
Source: Pediatr Rheumatol Online J. 2023 Jan 27;21:11. doi: 10.1186/s12969-022-00779-3 (PMC9881323; doi:10.1186/s12969-022-00779-3)
Supplement: Supplementary file 1 — Additional file 1: Supplement Table S1. The distribution of the ESSDAI score for each domain of 39 patients with childhood-onset primary Sjögren’s syndrome. [file 12969_2022_779_MOESM1_ESM.docx]

Supplement Table S1 The distribution of the ESSDAI score for each domain of 39 patients with childhood-onset primary Sjögren’s syndrome

| Patient | Domain | | | | | | | | | | | | Total | Therapy | | | | Follow-up of ESSDAI |
| --- | --- | --- | --- | --- | --- | --- | --- | --- | --- | --- | --- | --- | --- | --- | --- | --- | --- | --- |
|  | Constitutional | Lym | Glandular | Articular | Cutaneous | Pulmonary | Renal | Muscular | PNS | CNS | Hematological | Biological |  | Therapeutic regime | | GC tapering | |  |
| 1 | 0 | 4 | 0 | 0 | 3 | 0 | 0 | 0 | 0 | 0 | 0 | 0 | 7 | HCQ | | NE | | 1 |
| 2 | 0 | 0 | 0 | 0 | 0 | 0 | 0 | 0 | 0 | 0 | 0 | 2 | 2 | GC+HCQ | | NE^4)^ | | NE^4)^ |
| 3 | 0 | 0 | 0 | 0 | 6 | 0 | 0 | 0 | 0 | 0 | 0 | 1 | 7 | GC+HCQ | | <5mg/d^5)^ | | 0 |
| 4 | 0 | 0 | 0 | 0 | 3 | 0 | 0 | 0 | 0 | 0 | 0 | 1 | 4 | GC+HCQ+MMF | | <5mg/d^5)^ | | 0 |
| 5 | 6 | 0 | 0 | 2 | 0 | 0 | 0 | 0 | 0 | 0 | 0 | 1 | 9 | HCQ | | NE | | 1 |
| 6 | 0 | 0 | 0 | 0 | 0 | 0 | 15 | 0 | 0 | 0 | 0 | 2 | 17 | GC+HCQ→+MMF^2)^ | | Withdrawal of GC | | 0 |
| 7 | 0 | 0 | 4 | 0 | 0 | 0 | 0 | 0 | 0 | 0 | 0 | 2 | 6 | GC+HCQ | | Withdrawal of GC | | 0 |
| 8 | 3 | 0 | 0 | 2 | 0 | 0 | 0 | 0 | 0 | 0 | 0 | 2 | 7 | GC+HCQ | | NE^6)^ | | NE^6)^ |
| 9 | 0 | 4 | 2 | 0 | 0 | 0 | 0 | 0 | 0 | 0 | 0 | 2 | 8 | GC+HCQ→+MMF^1)^ | | Withdrawal of GC | | 2 |
| 10 | 0 | 0 | 0 | 4 | 6 | 0 | 0 | 0 | 0 | 0 | 0 | 2 | 12 | GC+HCQ+MMF | | NE^4)^ | | NE^4)^ |
| 11 | 3 | 0 | 0 | 0 | 3 | 0 | 0 | 0 | 0 | 0 | 2 | 2 | 10 | GC+HCQ | | Withdrawal of GC | | 2 |
| 12 | 6 | 0 | 0 | 0 | 9 | 0 | 0 | 0 | 0 | 0 | 6 | 0 | 21 | GC+HCQ | | <5mg/d^5)^ | | 1 |
| 13 | 0 | 0 | 0 | 0 | 0 | 0 | 5 | 0 | 0 | 0 | 0 | 2 | 7 | GC+HCQ | | NE^4)^ | | NE^4)^ |
| 14 | 3 | 0 | 0 | 0 | 0 | 0 | 0 | 0 | 0 | 0 | 0 | 0 | 3 | GC+HCQ | | Withdrawal of GC | | 0 |
| 15 | 0 | 0 | 0 | 0 | 0 | 0 | 5 | 0 | 0 | 0 | 0 | 0 | 5 | GC+HCQ | | Withdrawal of GC | | 5 |
| 16 | 0 | 4 | 0 | 0 | 0 | 0 | 15 | 0 | 10 | 0 | 0 | 2 | 31 | GC+HCQ+CYC→MMF^3)^ | | <5mg/d^5)^ | | 5 |
| 17 | 0 | 4 | 2 | 0 | 0 | 0 | 0 | 0 | 0 | 0 | 0 | 1 | 7 | GC+HCQ→+MMF^1)^ | | <5mg/d^5)^ | | 2 |
| 18 | 0 | 0 | 2 | 0 | 3 | 0 | 0 | 0 | 0 | 0 | 0 | 2 | 7 | GC+HCQ+MMF | | Withdrawal of GC spontaneously | | 1 |
| 19 | 0 | 0 | 0 | 0 | 3 | 0 | 0 | 0 | 0 | 0 | 0 | 1 | 4 | GC+HCQ+MMF | | Withdrawal of GC | | 2 |
| 20 | 0 | 0 | 2 | 0 | 0 | 0 | 0 | 0 | 0 | 0 | 0 | 0 | 2 | GC+HCQ | | <5mg/d^5)^ | | 0 |
| Patient | Domain | | | | | | | | | | | | Total | Therapy | | | Follow-up of ESSDAI | |
|  | Constitutional | Lym | Glandular | Articular | Cutaneous | Pulmonary | Renal | Muscular | PNS | CNS | Hematological | Biological |  | Therapeutic regime | GC tapering | |  |  |
| 21 | 0 | 0 | 0 | 0 | 9 | 0 | 0 | 0 | 0 | 0 | 6 | 0 | 15 | GC+HCQ | <5mg/d^5)^ | | 0 | |
| 22 | 0 | 0 | 0 | 0 | 6 | 0 | 0 | 0 | 0 | 0 | 0 | 1 | 6 | HCQ | NE^6)^ | | NE^6)^ | |
| 23 | 0 | 0 | 0 | 0 | 6 | 0 | 0 | 0 | 0 | 0 | 0 | 2 | 8 | GC+HCQ | NE^6)^ | | NE^6)^ | |
| 24 | 0 | 0 | 0 | 0 | 0 | 0 | 5 | 0 | 0 | 0 | 0 | 2 | 7 | GC+HCQ→+CYC→MMF^1)^ | <5mg/d^5)^ | | 0 | |
| 25 | 6 | 0 | 0 | 0 | 0 | 0 | 0 | 0 | 0 | 0 | 4 | 2 | 12 | GC+HCQ→+MMF^2)^→Tofacitinib^1)^ | Withdrawal of GC | | 2 | |
| 26 | 3 | 0 | 0 | 0 | 0 | 0 | 0 | 0 | 0 | 0 | 0 | 0 | 3 | GC+HCQ+MMF | <5mg/d^5)^ | | 0 | |
| 27 | 3 | 4 | 0 | 0 | 0 | 0 | 0 | 0 | 0 | 0 | 0 | 1 | 8 | GC+HCQ→+MMF^1)^ | Withdrawal of GC | | 0 | |
| 28 | 6 | 4 | 2 | 0 | 0 | 5 | 0 | 0 | 0 | 0 | 4 | 2 | 23 | GC+HCQ+CYC→MMF^3)^ | Withdrawal of GC | | 0 | |
| 29 | 0 | 8 | 2 | 0 | 0 | 0 | 0 | 0 | 0 | 0 | 0 | 1 | 11 | GC+HCQ+MMF | Withdrawal of GC | | 0 | |
| 30 | 0 | 0 | 0 | 0 | 0 | 0 | 0 | 0 | 0 | 0 | 6 | 0 | 6 | GC+HCQ | NE^6)^ | | NE^6)^ | |
| 31 | 0 | 0 | 0 | 0 | 6 | 0 | 0 | 0 | 0 | 0 | 6 | 1 | 13 | GC+HCQ→+MMF^1)^ | Withdrawal of GC | | 0 | |
| 32 | 0 | 4 | 0 | 0 | 6 | 0 | 0 | 0 | 0 | 0 | 2 | 1 | 13 | GC+HCQ+MMF | <5mg/d^5)^ | | 0 | |
| 33 | 0 | 0 | 0 | 2 | 0 | 5 | 0 | 0 | 0 | 0 | 0 | 1 | 8 | GC+HCQ+CYC→MMF^3)^ | <5mg/d^5)^ | | 4 | |
| 34 | 0 | 0 | 0 | 0 | 3 | 0 | 0 | 0 | 0 | 0 | 0 | 1 | 4 | GC+HCQ+MMF | <5mg/d^5)^ | | 3 | |
| 35 | 0 | 0 | 0 | 0 | 0 | 0 | 0 | 0 | 0 | 0 | 6 | 2 | 8 | GC+HCQ+MMF | <5mg/d^5)^ | | 0 | |
| 36 | 0 | 0 | 0 | 0 | 0 | 5 | 0 | 0 | 0 | 0 | 4 | 2 | 11 | GC+HCQ+MMF | <5mg/d^5)^ | | 0 | |
| 37 | 0 | 0 | 0 | 0 | 0 | 0 | 0 | 0 | 0 | 0 | 0 | 2 | 2 | GC+HCQ+MMF | Withdrawal of GC | | 0 | |
| 38 | 6 | 8 | 4 | 0 | 0 | 0 | 0 | 0 | 0 | 0 | 0 | 1 | 19 | GC+HCQ+MMF | <5mg/d^5)^ | | 0 | |
| 39 | 6 | 0 | 0 | 0 | 0 | 0 | 0 | 0 | 0 | 0 | 6 | 2 | 14 | GC+HCQ+CsA→MMF | <5mg/d^5)^ | | 0 | |

Lym: Lymphadenopathy and lymphoma, PNS: peripheral nervous system, CNS: central nervous system, NA:not applicable, GC: Glucocorticoid, HCQ: Hydroxychloroquine, MMF: Mycophenolate mofetil, CYC: Cyclophosphamide, CsA: Cyclosporine A, SLE: Systemic lupus erythematosus.

1. disease recurred under prescribed withdrawal of GC, 2) disease recurred after spontaneous withdrawal of GC, 3) MMF as maintenance therapy to CYC, 4) develop SLE, 5) Maintained with small dose of GC, 6) Loss to follow-up
