# Supplementary material for: Childhood-onset primary Sjögren’s syndrome in a tertiary center in China: clinical features and outcome
Source: Pediatr Rheumatol Online J. 2023 Jan 27;21:11. doi: 10.1186/s12969-022-00779-3 (PMC9881323; doi:10.1186/s12969-022-00779-3)
Supplement: Supplementary file 2 — Additional file 2. [file 12969_2022_779_MOESM2_ESM.pdf]

Editing Certificate

This document certifies that the manuscript

Childhood-onset primary Sjögren's syndrome in a tertiary center in China: Clinical features and outcome

prepared by the authors

Yinv Gong, Haimei Liu, Guomin Li, Tao Zhang, Yifan Li, Wanzhen Guan, Qiaoqian Zeng, Qianying Lv, Xiaomei Zhang, Wen Yao, Yu Shi, Hong Xu, Li Sun

was edited for proper English language, grammar, punctuation, spelling, and overall style by one or more of the highly qualified native English speaking editors at AJE.

This certificate was issued on **December 1, 2022** and may be verified on the AJE website using the verification code **9C5D-51A3-B2DE-618F-96EE**.

Neither the research content nor the authors' intentions were altered in any way during the editing process. Documents receiving this certification should be English-ready for publication; however, the author has the ability to accept or reject our suggestions and changes. To verify the final AJE edited version, please visit our verification page at aje.com/certificate. If you have any questions or concerns about this edited document, please contact AJE at support@aje.com.

AJE provides a range of editing, translation, and manuscript services for researchers and publishers around the world. For more information about our company, services, and partner discounts, please visit aje.com.
